# Non-specific low back pain: cross-sectional study of 11,423 children and youth and the association with the perception of heaviness in carrying of schoolbags

**DOI:** 10.7717/peerj.11220

**Published:** 2021-05-04

**Authors:** Agnieszka Kędra, Magdalena Plandowska, Przemysław Kędra, Dariusz Czaprowski

**Affiliations:** 1 Faculty of Physical Education and Health, Jozef Pilsudski University of Physical Education in Warsaw, Biala Podlaska, Poland; 2 Physiotherapy Unit, Department of Health Sciences, Poznan University of Medical Sciences, Poznan, Poland; 3 Department of Health Sciences, Olsztyn University, Olsztyn, Poland

**Keywords:** Low back pain, Pain, Children, Adolescent, Schoolbag

## Abstract

**Background:**

Carrying a schoolbag is a daily activity for most children and adolescents.****The perception of a schoolbag’s weight depends upon the individual and is a relevant theme in schoolchildren. Describing the association between the perception of heaviness in carrying a schoolbag and NLBP can facilitate the planning of preventive programs, quite different from those based on specific weight limits for schoolbags in children and youth.

**Objective:**

To determine the prevalence of non-specific low back pain (NLBP) and to analyse the association between the perception of heaviness in carrying of schoolbags and NLBP in Polish children and youth aged 10–19.

**Methods:**

This study included 11,423 children and youth (6,252 girls and 5,171 boys) from Poland. An original questionnaire was used to assess NLBP prevalence.

**Results:**

Among 11,423 of the respondents, 41.5% of the respondents admitted that they had experienced NLBP in the period of the last 12 months. The percentage of individuals reporting NLBP increased with age of participants, *p* < 0.001. Girls reported NLBP more often than boys (*p* < 0.001). Students with NLBP declared that their school backpack was heavy more often than students without LBP. Students who thought that their backpacks weighed too much manifested a 1.44 (95% Cl [1.33–1.55]) times higher probability to experience NLBP.

**Conclusion:**

In the examined group of schoolchildren a frequent occurrence of NLBP (41.5%) was noted. Its occurrence was related to female sex and age. Students who thought that their backpacks weighed too much manifested higher probability to develop NLBP.

## Introduction

Low back pain (LBP) has been defined as a public health issue. LBP experienced in childhood and adolescence is a factor increasing the risk of its occurrence in adult life (Siivola et al., 2004; Hestbaek et al., 2006). The majority of LBP cases are of non-specific character (Balagué et al., 2012; Minghelli, Oliveira & Nunes, 2014). Non-specific low back pain (NLBP) is defined as pain and discomfort located below the costal margin and above the inferior gluteal folds, with or without leg pain, not attributed to recognisable, known specific pathology (Burton et al., 2006).

Carrying a schoolbag is a daily activity for most children and adolescents. Some biomechanical studies suggest that the backpack carriage was associated with changes in body posture and with some biomechanical alterations in walking among children and adults (Devroey et al., 2007; Mackie & Legg, 2007; Liew, Morris & Netto, 2016). Previous studies have investigated the association between BP and schoolbag characteristics such as weight, type (design), method of carrying (Yamato et al., 2018; Calvo-Muñoz et al., 2020). However, the evidence has been inconsistent (Yamato et al., 2018). Some studies suggested that student‘s perception that the weight of the schoolbag is excessive for them may increase the risk of back pain (Dockrell, Simms & Blake, 2015; Dockrell, Blake & Simms, 2016). Students’ perception that the weight of the schoolbag is excessive for them, irrespective of its actual weight, is one of the non-mechanical factors. A study by Dockrell, Simms & Blake (2015) found that 49.8% of children were correct in the estimation of the weight of their schoolbag, 42.8% under-estimated and 7.4% over-estimated. The perception of schoolbag weight depends on the individual and is a relevant theme in schoolchildren.

Therefore, this study aimed to determine the prevalence of NLBP and to analyse the association between the perception of heaviness in carrying of schoolbags and LBP in Polish school-age children and youth aged 10–19.

## Material & Methods

### Participants

The study included Polish children and youth aged from 10–19 years. To determine the size of the sample the formula was used: 10% of all students in selected major cities from eastern Poland. The sample size for the group was calculated from at least 9000 individuals, plus an extra 20% to account for expected loses, for a total of 10,800 children and youth (Suresh & Chandrashekara, 2012). A 2-stage sampling method was adopted to obtain a representative sample of schoolchildren. In the first stage, public schools from three levels of education (primary, lower-secondary and upper-secondary schools) were randomly selected from ten major cities from eastern Poland. In the second stage, particular classes were selected (stratified phase sampling) (Brown et al., 1999). Ultimately, the study included students from the 4th to 6th grade of primary school (children aged 10–13; mean body weight: 43.7 kg, mean height: 1.55 m), the 1st to 3rd grade of lower-secondary school (14–16 years; mean body mass: 56.3 kg, mean height: 1.68 m) and the 1st to 3rd grade of upper-secondary school (17–19 years; mean body mass: 62.2 kg, mean height: 1.72 m).

11619 participants accepted to respond to the questionnaire. Of those that responded, 196 were excluded for incomplete questionnaires. The final analysis included 11,423 questionnaires (6,252 from girls and 5,171 from boys), which constitutes 98.3% of the total number of participants.

This was a cross-sectional study, approved by the Senate Research Ethics Committee (no. SKE 01-31/2012). The study was conducted in accordance with the Declaration of Helsinki. All the participants and their parents or guardians gave written informed consent.

### Questionnaire

A questionnaire was applied as a research tool (File S2) (Kędra et al., 2019). The questionnaire was completed in the presence of one of the authors of the paper. While the participants filled out the survey, they were able to ask the author if they had any problems with the questionnaire. The survey was anonymous and voluntary.

The first part of the questionnaire was addressed to all respondents and concerned the following aspects: sex and date of birth, the perception of heaviness in carrying of schoolbag and information about experiencing LBP during the last year (12 months). Students who gave a negative response to this question about experiencing LBP did not complete the remaining part of the questionnaire. Individuals who declared that they had experienced LBP during the last year (12 months) answered the questions in the second part. The second part of the questionnaire included questions regarding the frequency of LBP, types of situations in which LBP occurred or increased, ways of coping with pain and suggestions of ways of reducing LBP. The region of LBP was explicitly described through a diagram in the questionnaire.

LBP was assessed using the following question: “Have you experienced low back pain within the last year (12 months)?”. The answer options were: “no”, “yes”, “yes, but only during menstruation”. “Yes” was considered to denote the presence of LBP.

The reliability of the questionnaire was assessed. The Kappa coefficient value for all the analysed variables was equal to or higher than 0.91 (Kędra et al., 2019).

After consulting the school doctor, students with such spinal diseases which may cause LBP as Scheuermann’s disease, spondylolysis, spondylolisthesis, rheumatic diseases, tumours, sarcomas^5^ were excluded from the study (23 students). In the case of the girls, those who experience LBP only during menstruation were also excluded.

### Statistical analysis

Parameters were described using basic measurements of descriptive statistics for qualitative variables, i.e., percentage. Chi-square test was used to comparison of the prevalence of LBP taking into account sex and age. The association between the perception of heaviness in carrying of schoolbag and LBP outcome was analysed by univariate logistic regression with the odds ratio (OR) and 95% confidence interval (CI). Statistical significance was set at *p* < 0.05. The collected material was organised and analysed with the use of Statistica 13.3 calculation software by Statsoft (Poland).

## Results

### Prevalence of NLBP

Among 11,423 of the respondents, 4,743 (41.5%) declared that they had experienced NLBP in the period of the last 12 months. Girls reported NLBP more often than boys (49.5% vs. 31.8%) (*p* < 0.001). The percentage of individuals reporting NLBP increased with age and this difference was statistically significant, *p* < 0.001 (Table 1).

**Table 1:** The prevalence of NLBP with regard to sex and age of the respondents and significance from the Chi-square test.

	All %	with NLBP %	without NLBP %	*p* value
All	100	41.5	58.5	–
Sex				
Girls	54.7	49.5	50.5	<0.001
Boys	45.3	31.8	68.2	
Age				
10–11 years	10.4	13.2	86.8	<0.001
12–13 years	15.4	23.2	76.8	
14–15 years	28.7	36.5	63.5	
16–17 years	24.8	54.0	46.0	
18–19 years	20.7	61.4	38.6	

**Notes.**

NLBP, non-specific low back pain.

Statistical significance was set at *p* < 0.05.

### Prevalence and circumstances of NLBP

The largest group was constituted by the respondents who experienced NLBP very rarely, i.e., 1–2 times a year (49.8% of the respondents). The analysis of the prevalence of NLBP with regard to sex revealed that boys declared rare pain (1–2 times a year) more often than girls (54.3% vs. 47.4%) (Table 2).

**Table 2:** The prevalence, circumstances of NLBP, and ways of dealing with NLBP in the group of students divided according to sex (*n* = 4743).

	NLBP (*n* = 4743)	Sex	
		Girls (*n* = 30,97)	Boys (*n* = 1,646)
	%	%	%
NLBP frequency			
Very rare NLBP (1–2/year)	49.8	47.4	54.3
NLBP a few times a year (3–6/year)	38.4	39.7	35.7
Frequent or constant NLBP (more than 1–2 months)	11.9	12.9	10.0
Circumstances in which NLBP occurred^a^			
Lifting heavy objects	53.2	58.2	43.9
Carrying a school backpack	39.9	43.2	33.6
Physical exercises	9.5	8.2	11.9
PE lesson	19.8	20.9	17.6
Sitting	49.7	50.6	47.9
Mental stress	8.6	10.0	6.0
Changeable weather	10.1	10.9	8.7
Other	28.3	37.6	10.8
Seeking doctor’s help			
Yes	18.4	19.5	16.5
No	81.6	80.5	83.5
Ways of coping with NLBP^a^			
Medicines prescribed by a doctor	4.9	5.9	2.9
Generally available painkillers	16.1	19.8	9.2
Electrotherapy procedures	20.9	22.6	17.6
Physiotherapeutic treatment other than electrotherapy (gymnastics, exercises)	41.2	40.4	42.8
Rest	68.3	70.8	63.6
Other	5.3	4.4	7.0
What can reduce NLBP, according to the students^*^			
I don’t know	6.3	4.8	9.2
Increased physical activity	44.7	44.0	45.9
Limiting the weight of a school backpack	66.3	73.3	53.1
Reducing the number of hours spent in a sedentary position	42.9	46.2	36.8
Adapting school equipment (desk, chair) to body height	30.1	30.4	29.5
Increasing the availability of painkillers	3.6	3.3	4.1
Other	3.1	2.7	3.8

**Notes.**

NLBP- non-specific low back pain.

a The numbers do not add to 100% since the respondents were allowed to choose more than one answer.

It can be noted that in the age groups of 10–13-year-olds and 14–16-year-olds, very rare pain (1–2 times a year) was reported more often than in the group of 17–19-year-olds (52.7% and 52.8% vs. 47.3%). In turn, the percentage of students reporting frequent or constant pain (occuring 1–2 times a month) was higher in the group of 17–19-year-olds than in the groups of 10–13 and 14–16-year-olds (12.4% vs. 11.3% and 11.3%) (Table 3).

**Table 3:** The prevalence, circumstances of NLBP, and ways of dealing with NLBP in the group of students divided according to age (*n* = 4743).

	Age		
	10–13 years (*n* = 564)	14–16 years (*n* = 1,552)	17–19 years (*n* = 2,627)
	%	%	%
NLBP frequency			
Very rare NLBP (1–2/year)	52.7	52.8	47.3
NLBP a few times a year (3–6/year)	36.0	35.9	40.3
Frequent or constant NLBP (more than 1–2 months)	11.3	11.3	12.4
Circumstances in which NLBP occurred^*^			
Lifting heavy objects	58.9	53.9	51.7
Carrying a school backpack	44.7	41.8	37.7
Physical exercises	7.5	8.4	10.6
PE lesson	21.6	21.8	18.2
Sitting	44.2	46.1	53.0
Mental stress	8.0	9.0	8.6
Changeable weather	7.4	8.2	11.8
Other	21.3	25.4	31.5
Seeking doctor’s help			
Yes	23.2	18.9	17.1
No	76.8	81.1	82.9
Ways of coping with NLBP^*^			
Medicines prescribed by a doctor	6.4	4.1	5.0
Generally available painkillers	10.6	13.3	18.9
Electrotherapy procedures	19.7	21.3	20.9
Physiotherapeutic treatment other than electrotherapy (gymnastics, exercises)	41.3	49.5	42.2
Rest	60.5	64.1	72.4
Other	6.4	7.1	4.0
What can reduce NLBP, according to the students^*^			
I don’t know	12.2	6.5	4.9
Increased physical activity	33.0	39.8	50.1
Limiting the weight of a school backpack	56.9	68.0	67.3
Reducing the number of hours spent in a sedentary position	34.0	35.2	49.4
Adapting school equipment (desk, chair) to body height	27.8	28.4	31.6
Increasing the availability of painkillers	4.3	3.6	3.4
Other	6.2	4.3	1.8

**Notes.**

NLBP, non-specific low back pain.

* The numbers do not add to 100% since the respondents were allowed to choose more than one answer.

While analysing the circumstances in which NLBP occurs with regard to the respondents’ sex, it can be concluded that both in the group of girls and among boys, the most common circumstances included lifting heavy objects (girls 58.2%, boys 43.9%), sitting (girls 50.6%, boys 47.9%), and carrying a school backpack (girls 43.2%, boys 33.6%) (Table 2). In the case of all the age groups (10–13 years, 14–16 years, 17–19 years), a considerable group of students declared that they felt NLBP during PE lessons (21.6% vs. 21.8% vs. 18.2%) (Table 3).

### Ways of coping with NLBP

Rest (68.3%) and physiotherapeutic treatment other than electrotherapy (41.2%) were the most common ways of coping with NLBP in all respondents. The results indicate that an inconsiderable group of students (18.4%) sought doctor’s help due to NLBP (Table 2). The percentage of students seeking doctor’s help due to NLBP decreases with age (23.2% vs. 18.9% vs. 17.7%) (Table 3).

Over 16% of the respondents cope with NLBP by taking generally available painkillers. It can be noted that with age, the percentage of students who take painkillers to mitigate NLBP increases (10.6% vs. 13.3% vs. 18.9%) (Tables 2 and 3).

The respondents experiencing NLBP were asked what, according to them, can reduce the pain. The largest group was comprised of respondents who replied “Limiting the weight of a school backpack” (66.3%) and those who suggested increasing the level of physical activity (44.7%). According to a considerable group of students (42.9%), reducing the number of hours spent in a sedentary position might reduce NLBP (Table 2).

**Table 4:** The prevalence of NLBP in students with regard to the perception of heaviness in carrying of schoolbags and students sex and age (*n* = 11,423).

Perception of heaviness in carrying of schoolbags			All %	NLBP	
				with NLBP %	without NLBP %
All		Heavy	55.4	60.6	51.7
		Adequate	44.6	39.4	48.3
Sex	Girls				
		Heavy	61.5	65.2	57.9
		Adequate	38.5	34.8	42.1
	Boys				
		Heavy	48.1	52.1	46.2
		Adequate	51.9	47.9	53.8
Age	10–11 years				
		Heavy	43.5	50.0	42.5
		Adequate	56.5	50.0	57.5
	12–13 years				
		Heavy	50.4	59.1	47.8
		Adequate	49.6	40.9	52.3
	14–15 years				
		Heavy	58.6	61.5	56.9
		Adequate	41.4	38.5	43.1
	16–17 years				
		Heavy	58.2	61.1	54.7
		Adequate	41.8	38.9	45.3
	18–19 years				
		Heavy	57.4	61.0	51.8
		Adequate	42.6	39.0	48.2

**Notes.**

NLBP, non-specific low back pain.

**Table 5:** Association between the perception of heaviness in carrying of schoolbags and NLBP.

		Crude OR	95%CI	*p* value
All		1.44	1.330–1.55	<0.001
Sex				
	Girls	1.36	1.23–1.51	<0.001
	Boys	1.27	1.13–1.43	<0.001
Age				
	10–11 years	1.35	0.96–1.89	0.081
	12–13 years	1.58	1.26–1.98	<0.001
	14–15 years	1.21	1.05–1.40	<0.01
	16–17 years	1.30	1.16–1.47	<0.001
	18–19 years	1.45	1.23–1.72	<0.001

**Notes.**

NLBP non-specific low back pain OR odds ratio CI confidence intervals

The association between the perception of heaviness in carrying of schoolbags (no = 0, yes = 1) and NLBP (no = 0, yes = 1) was analysed by univariate logistic regression with the calculation of the corresponding OR and 95% CI. Statistical significance was set at *p* < 0.05.

### The prevalence of NLBP with regard to their perception of heaviness in carrying of schoolbags

Students (with and without NLBP) who declared that their school backpack was heavy constituted the highest percentage (55.4%). Students with NLBP declared that their school backpack was heavy more often than students without NLBP. Such a situation was noted in both sex groups and in all age groups. Girls with NLBP declared that their school backpack was heavy more often than boys with NLBP (65.2% vs. 52.1%). The analysis revealed that up to the age of 15, the percentage of students declaring that their school backpack was heavy was increasing (43.5% vs. 50.4% vs. 58.6%), and from the age of 16 was gradually decreasing (Table 4).

The perception of heaviness in carrying of schoolbags was a significant factor for NLBP. Students who thought that their backpacks weighed too much manifested a 1.44 times higher probability (95% Cl [1.33–1.55]) to develop NLBP. Girls who declared that their school backpack was heavy presented 1.36 times greater probability (95% Cl [1.23–1.51]) to develop NLBP than girls who declared that their school backpack was adequate. Boys manifested a 1.27 times higher probability (95% Cl [1.13–1.43]) to develop NLBP. In each age group, the declared heavy schoolbag is a risk factor for NLBP (Table 5).

## Discussion

The aim of the research was to determine the prevalence of NLBP and to analyse the association between the perception of heaviness in carrying of schoolbag with LBP in Polish school-age children and youth aged 10-19. This study showed that among 11,423 of the respondents, 41.5% of the respondents admitted that they had experienced NLBP in the period of the last 12 months, and the percentage of individuals reporting NLBP increased with age. Girls reported NLBP more often than boys. Students who thought that their backpacks weighed too much manifested a 1.44 times higher probability (95% Cl [1.33–1.55]) to develop NLBP.

Our study revealed that NLBP (in the period of the last 12 months) was experienced by a considerable group (41.5%) of children and youth aged 10-19, and the percentage of students experiencing NLBP increased with age. Studies by other authors reported the prevalence of NLBP among children and adolescents is from 0.8 to 84% (Jeffries, Milanese & Grimmer-Somers, 2007; Calvo-Munoz, Gomez-Conesa & Sanchez-Meca, 2013; Minghelli, Oliveira & Nunes, 2014; Bento et al., 2020). Differences regarding the percentage of students experiencing LBP may be caused by different study designs, different methods of data collection, different age groups taking part in the studies or different definitions of LBP. Differences in percentage values may result from the fact that different periods of experiencing LBP were taken into account.

Our study revealed that girls reported NLBP more often than boys (49.5% vs. 31.8%). A higher percentage of LBP among girls than boys was also noted in the studies by other authors (Kovacs et al., 2003; Oksuz, 2006). It is implied that a higher prevalence of LBP among women is caused by different pain thresholds, differences in pain perception between sexes (Negrini & Carabalona, 2002; Burton et al., 2006) and by pubertal growth, i.e., hormone-induced changes at puberty that may affect attitudes to or perception of pain (Burton et al., 2006).

The present study also showed that students with NLBP more often declared that their school backpack was heavy. Girls with NLBP declared that their school backpack was heavy more often than boys with NLBP. Students who claimed that their backpacks were too heavy were 1.44 times more likely to develop NLBP. Our study found that carrying a school backpack was one of the most common circumstances in which NLBP occurs. Moreover, the largest group replied that, according to them, “Limiting the weight of a school backpack” can reduce the pain.

Our analysis revealed that up to the age of 15, the percentage of students declaring that their school backpack was heavy was increasing and from the age of 16 was gradually decreasing. Regular activity involving loading of the spine may improve trunk musculature strength, endurance and tissue tolerance for load. Carrying a schoolbag provides a form of physical exercise and physical activity whilst carrying a school bag (in the form of walking or riding to school) appeared to offset prolonged exposure as a factor identifying spinal pain (Haselgrove et al., 2008). Studies showed that the majority of children carried their schoolbags for 20 min or less on the way to school and travelled to school by car (Dianat et al., 2013; Dockrell, Simms & Blake, 2015). Primary school children frequently remain in the same classroom during the day and consequently do not need to carry their schoolbags during school hours. In contrast, lower- and upper-secondary school children are more likely to move from one classroom to another between classes, according to their timetable, putting on and taking off their schoolbag as required and carrying them for longer periods of time (Dockrell, Simms & Blake, 2015). Moreover, regular activity involving loading of the spine may improve trunk musculature strength, endurance and tissue tolerance for load (Haselgrove et al., 2008).

Our study reported that students who thought that their backpacks weighed too much manifested higher probability to develop NLBP. To date, the evidence that aspects of schoolbags is associated with back pain is inconsistent. Some studies suggested that heavy schoolbags could be a risk factor contributing towards musculoskeletal diseases (Negrini & Carabalona, 1999; Dianat et al., 2014). On the other hand, a recent systematic review reported that the available evidence does not reveal a relationship between schoolbag weight and LBP among children and teenagers (Calvo-Muñoz et al., 2020). A systematic review by Yamato et al. (2018) found that schoolbag characteristics such as weight, design and carriage method do not increase the risk of developing back pain in children and youth, but there is some evidence that the perception of heaviness is associated with back pain (Yamato et al., 2018). Some studies suggested that student‘s perception that the weight of the schoolbag is excessive for them, irrespective of its actual weight, may increase the risk of back pain (Dockrell, Simms & Blake, 2015; Dockrell, Blake & Simms, 2016). The subjective perception of heaviness in carrying of schoolbag is a relevant theme in schoolchildren. Study by Haselgrove et al. (2008) showed that both the perceived weight and perceived fatigue during carriage of the school bag are significantly associated with back pain. Possible explanations for this include a diminished physical capacity (back strength, endurance) associated with LBP, rendering the same load relatively heavier for individuals with LBP compared with healthy individuals, or an increased tissue sensitivity to load associated with LBP (Haselgrove et al., 2008). Reporting the school bag to be heavy may indicate poor trunk muscle endurance and control which are known risk factors for back pain (Negrini & Carabalona, 2002). Future studies should attempt to explain the importance of physical and psychosocial factors related to the perception of load in schoolchildren.

### Study limitations

The presented research used recognised methods of assessing LBP (Vikat et al., 2000; Wedderkopp et al., 2009; Heneweer, Vanhees & Picavet, 2009), but the obtained results are a subjective assessment of the reported NLBP.

The results of this study should be interpreted with caution because of its cross-sectional design. This evidence of itself is insufficient, but evidence from cross-sectional studies support the findings from prospective studies (Yamato et al., 2018). Perception of schoolbag weight is one of many aspects of schoolbag usage. Therefore, it is important to consider other factors related to the method of carrying the schoolbag that can affect LBP.

### Study strengths

A large population sample (*n* = 11423) and a wide age range (10–19 years) constitute study strength as it makes it possible to illustrate the significance of the problem of NLBP in particular age groups. The reliability of the questionnaire applied in this study was assessed. The Kappa coefficient value for all the analysed variables was equal to or higher than 0.91, which indicates the reliability of information gathered with it. According to the authors’ knowledge, it is the first study regarding NLBP on such a large group of students from Poland. This study presents not only the frequency of pain, but also it describes an association between the perception of heaviness in carrying a schoolbag and LBP, which can facilitate the planning of preventive programs, quite different from those based on specific weight limits for schoolbags in children and youth.

## Conclusion

NLBP occurred frequently in Polish schoolchildren. Its occurrence was related to female sex and age. Students who thought that their backpacks weighed too much manifested higher probability to develop NLBP. Greater understanding of schoolbag related-factors for LBP is important to planning of preventive programs, quite different from those based on specific weight limits for schoolbags in children and youth.

##  Supplemental Information

10.7717/peerj.11220/supp-1 Supplemental Information 1 Raw data applied for data analyses

10.7717/peerj.11220/supp-2 Supplemental Information 2 Questionnaire (Polish version)

10.7717/peerj.11220/supp-3 Supplemental Information 3 English translation of the questionnaire

## Additional Information and Declarations

### Data Availability

The following information was supplied regarding data availability: The raw measurements are available in the Supplemental File.
