# Supplementary material for: Non-specific low back pain: cross-sectional study of 11,423 children and youth and the association with the perception of heaviness in carrying of schoolbags
Source: PeerJ. 2021 May 4;9:e11220. doi: 10.7717/peerj.11220 (PMC8103923; doi:10.7717/peerj.11220)
Supplement: Supplemental Information 2 [file peerj-09-11220-s002.pdf]

KWESTIONARIUSZ

Szanowni Państwo!

Zwracamy się z uprzejmą prośbą o udzielenie odpowiedzi na postawione niżej pytania. Ankieta jest anonimowa. Celem badań jest zgromadzenie informacji, na temat częstości występowania i czynników ryzyka bólu kręgosłupa. Wyniki wykorzystane zostaną wyłącznie do celów naukowych.

W pytaniach oznaczonych ***** *można wybrać więcej niż jedną odpowiedz, w pozostałych należy wybrać tylko jedną z zaproponowanych odpowiedzi.*

Zaznacz kółkiem wybraną przez Ciebie odpowiedź, np. (k)

Dziękujemy

Dzisiejsza data....................

Płeć: ☐ Kobieta / ☐ Mężczyzna **Pełna data urodzenia (dzień, miesiąc, rok): ....**

1. Odczuwam, że mój bagaż do szkoły jest:

a) bardzo ciężki
b) umiarkowanie ciężki
c) lekki
d) zależy w które dni

2. Czy odczywałeś/aś dolegliwości bólowe kręgosłupa w ostatnich 12 miesiącach?

a) nie
b) tak, rzadko (1-2/12 miesięcy)
c) tak, często (3-6 razy/12 miesięcy)
d) tak, bardzo często i ciągle (więcej niż 1-2 razy w miesiącu)
e) tylko w czasie miesiączki (menstruacji)- dotyczy dziewcząt

Osoby, które w powyższym pytaniu (nr 2) zaznaczyły odpowiedź „a) nigdy”, nie udzielają odpowiedzi na kolejne pytania. Dziękujemy!

3. Ból mojego kręgosłupa zlokalizowany jest w odcinku *:

a) szyjnym

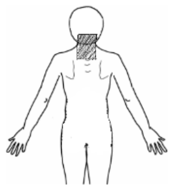

b) piersiowym

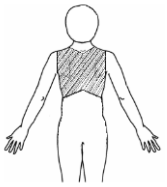

c) lędźwiowym

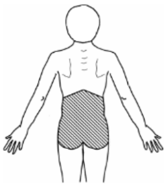

4. W jakich okolicznościach pojawia się ból kręgosłupa

a) podczas podnoszenia ciężkich przedmiotów
b) podczas noszenia plecaka szkolnego
c) podczas ćwiczeń fizycznych
d) na lekcji wychowania fizycznego
e) podczas siedzenia
f) podczas stresu psychicznego
g) zmiennej pogody
h) inne (jakie)...

5. Czy korzystasz z pomocy lekarza z powodu dolegliwości bólowych kręgosłupa?

a) tak
b) nie

6. Z bólami kręgosłupa radzę sobie przez:

a) przyjmowanie zaleconych przez lekarza leków
b) przyjmowanie leków dostępnych bez recepty
c) zabiegi w gabinecie fizykoterapii
d) zabiegi fizjoterapeutyczne inne niż fizykoterapia (gimnastyka, ćwiczenia zalecone przez fizjoterapeutę)
e) odpoczynek
f) inne (jakie)………

7. Jak myślisz, co może zmniejszyć ból kręgosłupa?

a) nie wiem
b) zwiększenie aktywności fizycznej
c) zmniejszenie ciężaru plecaka szkolnego
d) zmniejszenie liczby godzin spędzanych w pozycji siedzącej
e) dostosowanie sprzętu szkolnego (biurko, krzesło) do wysokości ciała
f) zwiększenie środków przeciwbólowych
g) inne (jakie)……
