# Supplementary material for: Non-specific low back pain: cross-sectional study of 11,423 children and youth and the association with the perception of heaviness in carrying of schoolbags
Source: PeerJ. 2021 May 4;9:e11220. doi: 10.7717/peerj.11220 (PMC8103923; doi:10.7717/peerj.11220)
Supplement: Supplemental Information 3 [file peerj-09-11220-s003.pdf]

QUESTIONNAIRE

For the purpose of the article the questionnaire was translated from Polish into English.

Dear Participants,

We would like to ask you to answer the following questions. The questionnaire is anonymous. The aim of the study is to collect information regarding the frequency of occurrence and risk factors of back pain. The results will be used only for scientific purposes.

*In the questions marked with * you may choose more than one answer, in the remaining questions only one answer can be selected.*

Thank you

Sex: Female/Male

Date of birth: day.....................month...........................year......................

1. The weight of your school backpack is:

a) very heavy
b) heavy
c) adequate
d) it depends on the days

2. Have you experienced back pain for the last year (12 months)?

a) no, never
b) yes, rarely (1-2 times/year)
c) yes, a few times a year (3-6 times/year)
d) yes, very often or constantly (more than 1-2 times/month)
e) yes, but only during menstruation

Individuals who chose answer ‘a) no, never’ in the above question (no 3), don’t answer the following questions. Thank you!

3. Back pain is located in:*

a) the cervical spine
b) the thoracic spine
c) the lumbar spine

4. In what circumstances does the back pain usually occur?*

a) while lifting heavy objects
b) while carrying a school backpack
c) during physical exercises
d) during PE

e) while sitting
f) during mental stress
g) during weather changes
h) other (specify)………

5. Do you seek doctor’s help?

a) Yes
b) No

6. How do you cope with back pain?

a) medicines prescribed by a doctor
b) generally available painkillers
c) electrotherapy procedures
d) physiotherapeutic treatment other than electrotherapy (gymnastics, exercises)
e) rest
f) other (specify)...

7. How do you think what can reduce back pain?

a) I don’t know
b) increased physical activity
c) limiting the weight of a school backpack
d) reducing the number of hours spent in a sedentary position
e) adapting school equipment (desk, chair) to body height
f) increasing the availability of painkillers
g) other (specify)...
